# Developing assessment flow for damage estimation of mud housing typology through a case study against coastal floods

**DOI:** 10.1038/s41598-023-33468-6

**Published:** 2023-04-25

**Authors:** N. Aishwarya, K. Aniruddha, D. Sutapa, H. A. Bharath

**Affiliations:** 1 Ranbir and Chitra Gupta School of Infrastructure Design and Management, Indian Institute of Technology Kharagpur, Kharagpur, West Bengal 721302 India; 2 Architecture, Town and Regional Planning, Indian Institute of Engineering Science and Technology, Howrah, West Bengal 721302 India

**Keywords:** Natural hazards, Sustainability

## Abstract

Rising instances of prolonged inundation due to climate-aggravated high tide flooding are economically burdensome for resource-crunched developing nations that bear enormous damage due to loss of built infrastructure (housing in this case). Regardless of the loss, coastal flood impact on buildings is rarely given precedence. The mud building typology in India captures 34% of existing housing demand primarily within ruralIndia; for instance, 75% of the housing in Sagar Island uses mud as the dominant construction material, making it an ideal case for the proposed research. The multivariate nature of damage and empirical data constraint associated with mud buildings propels the development of two unconventional damage assessment approaches using multivariate-probabilistic technique. The proposed literature-based approach uses logical reasoning based on the available scientific evidence whereas the lab-based approach uses the insights from structural analysis of scaled model. The damage matrix created from both the approaches are used to analyse a common flood data (depth & duration) generated using 1000 Montecarlo simulations. The resultant Damage Stage values confirm the versatility of either approach over spatial (local to regional)—temporal (flood character and intensity) dimensions. The lab-based approach proved to be a better alternative considering the availability of continuous records on damage behaviour and precise information on the flood threshold of dominant building material, a crucial component of the multivariate damage assessment process.

## Introduction

Coastal flooding due to high tide anomalies is calamitous, precisely due to prolonged inundation causing widespread destruction of livelihood, housing, and infrastructure in low-lying coastal areas^1–5^. The anomalies in this paper refers to the tidal water height that causes prolonged flooding within the low-lying coastal areas^6^. The spatial (geographical extent) and temporal (annual frequency) intensity of such high tides flooding are likely to enhance in the rapidly changing climate scenario that leads to an unprecedented rise in the mean sea level (MSL)^7,8^. South East Asia is among the major victims of MSL rise as it exposes around 300 million people (93 million in China, 42 million in Bangladesh, and 36 million in India) to potential risk of coastal flooding^9,10^. About 60 percent of this population located in low-elevation coastal zones of South Asia is rural and underequipped to invest in climate-resilient infrastructure^11^. Hence, the recurring floods would be economically burdensome for resource-crunched developing countries^12–16^.

India lost about 4.6 million US$ due to floods between 1953 and 2016, including the value of 1.2 million fully and partially damaged housing. The state of West Bengal accounts for 40% of the total housing loss^17^. The housing damage during cyclone Bulbul (2019), Aamphan (2020), and Yaas (2021) was approximated between 0.28 million, 2.86 million to 0.3 million houses in the affected district of West Bengal^18,19^. The loss of housing is critical as it affects mental, social, and economic well-being, inflicting chronic poverty and large-scale climate-induced displacements^20–24^. Regardless of the loss, coastal flood impact on buildings is rarely given precedence in developing countries due to its resource-intensive character, absence of aboriginal damage catalogues/damage records, and discreet behaviour based on building typology, preventing adaption of the foreign damage curves on native buildings.

For instance, little is known about the flood damage behaviour associated with mud housing. Mud buildings have displayed global dominance as vernacular construction style, fulfilling the housing requirements, particularly within developing countries, because of the climate benefits and associated affordability^25–27^. In India, mud housing, also referred to as non-engineered or kutcha housing, comprising about 34% of the total housing stock (Ministry of Housing and Urban Poverty Alleviation^28–30^, if integrated with disaster-resilient techniques, can relinquish the shortage of 18.78 million housing among the low-income groups^31,32^. Hence, investigation regarding the prevailing cost of damage is critical for investing in flood resilient housing schemes as it requires economic appraisals for optimal resource distribution within developing nations^33^.

### Damage behaviour in the case of mud buildings

In mud buildings, the damage behaviour depends on ‘prolonged inundation’ along with flood depth as a prominent reason for failure^28,34,35^. The convenience of interpreting depth values through visual inspections and measuring gauges in the post-hazard scenario has popularized its conduct over other flood characteristics in an empirical data analysis/processing^36–40^. Islam^41^ was the first to consider a multivariate approach for mud housings using depth and duration variables. The data used was synthetic, and the study clearly stated that the deterministic approach was inconsistent (actual damage data) in portraying the relationship between depth and duration variables.

Multivariate probabilistic damage models can handle this inconsistency reasonably well with their ability to integrate multiple flood characters and data variance in the estimation process^39^. Unlike empirical, the synthetically manifested data using the probabilistic model can be extrapolated over spatial (local to regional) and temporal dimensions (flood character and intensity), allowing pragmatic application in the data-scarce region^42,43^. However, estimation, calibration, and validation of the probable loss in response to the synthetic damage data require reliable knowledge of the mud housing damage behaviour(fragility function or curve). Its absence which is also referred to as predefined Damage Stages (DS) in the case of mud housing, limits the ability of the research community and local authorities to estimate the total cost of flood damage. The predefined damage curves suggest the probable loss in response to ‘the degree of physical damage’ incurred when the building is in direct contact with floodwater. It is represented on a scale of 0 (no loss) to 1 (total loss) on the Y-axis with respective flood intensity on X-axis. The loss is correlated to the monetary liabilities required to repair or reconstruct building components. The damage matrix is not to be confused with damage curves in this paper as it discusses the behavioural aspect of one (particular) building that can generically define the damage behaviour of its entire typology in the absence of observed data^44–47^. Therefore, this research aims to propose two distinct approaches (literature and lab-based approach) based on multivariate-probabilistic technique for evaluating the mud building damage behaviour, applicable in data-scarce region.

**Figure 1:**
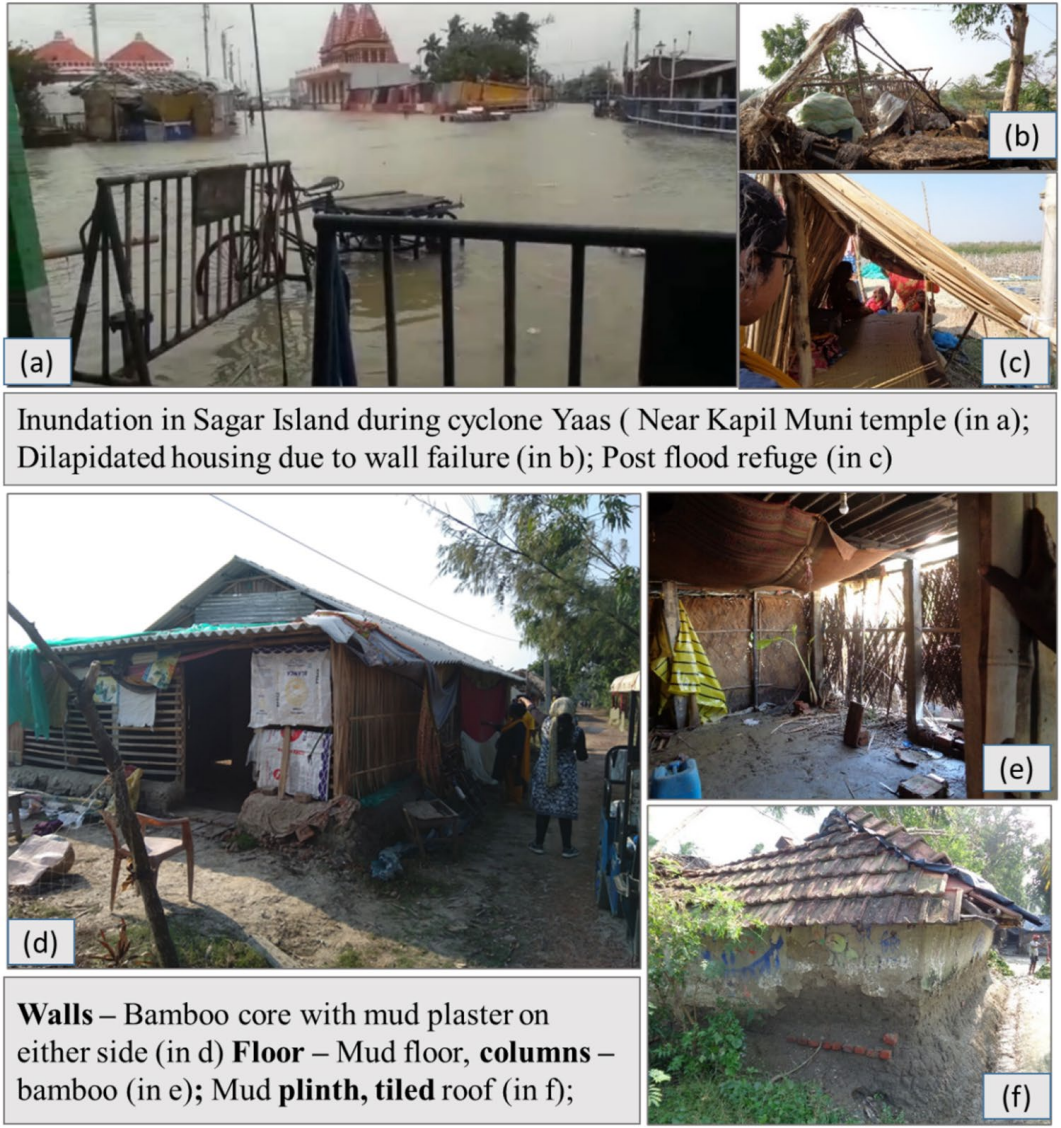
Inundation, post inundation scenario along with primary housing typology at Sagar.

**Figure 2:**
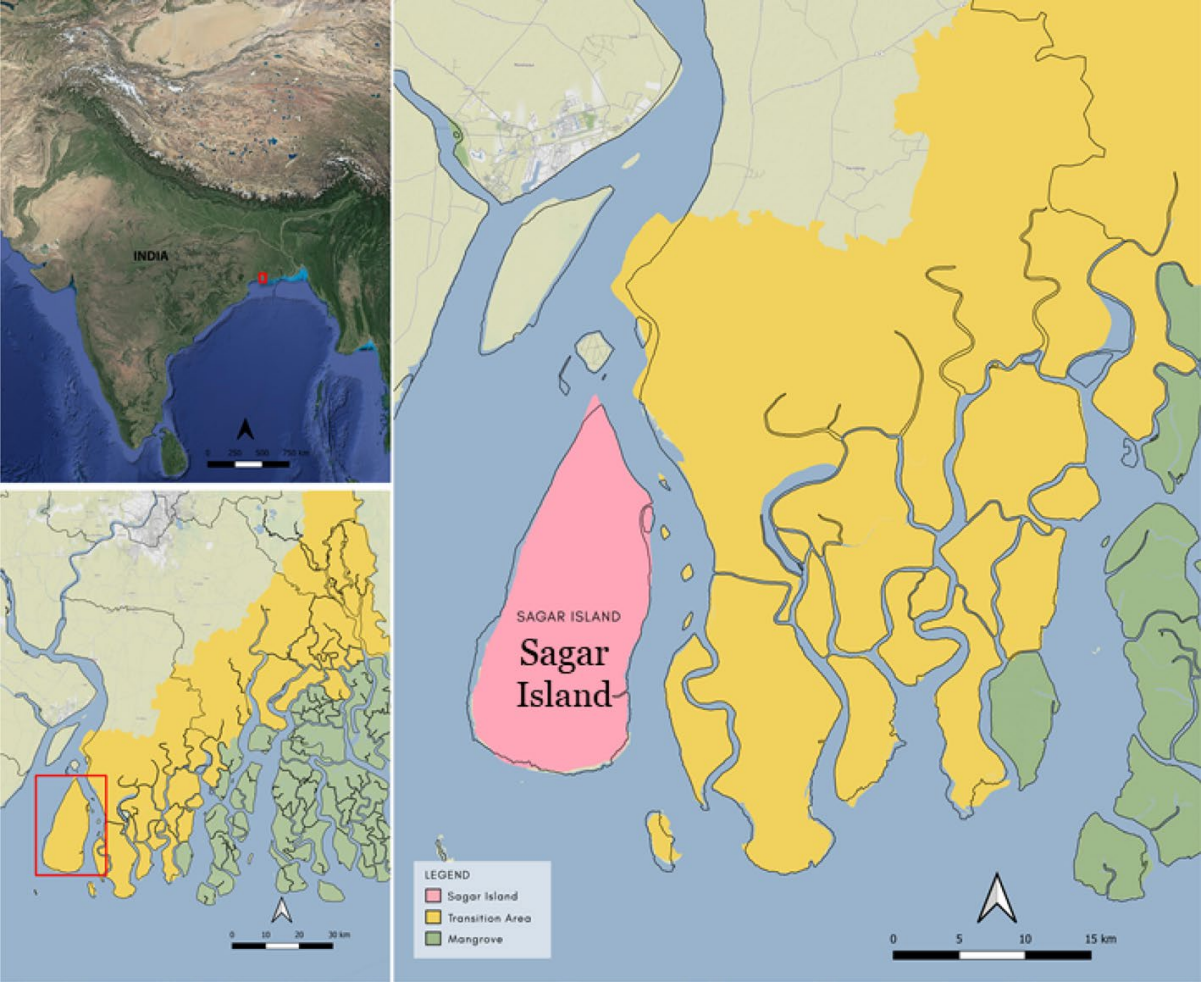
Study area—Sagar Island (West Bengal, India).

The model proposed uses the ‘wattle and daub’ construction technique for its depiction. It is a dominant housing typology practiced among the densely populated rural settlements in the coastal district of West Bengal, India. The coastal Bengal belongs to the habited part of the largest mangrove forest Sundarbans—the United Nations Educational, Scientific and Cultural Organization (UNESCO) heritage site cleared in the early 1800s for cultivation. The region is highly vulnerable to slow-rising coastal floods, which have become more common in the past ten years. Figure 1 presents a glimpse of the post-flood situation of affected regions, primarily Sagar Island. The Island, with an area of about 282 sq. km (in Fig. 2) is located between 21.6276° N to 21.8842° N and 88.0408° E to 88.1278° E in South 24 Paraganas, and has witnessed repeated incidents of high tidal anomalies^48,49^. The most pronounced flood incidents were recorded during cyclone Aila (2009) and the last three consecutive cyclones, Bulbul (2019), Aamphan (2020), and Yaas (2021). The Island saw an exponential growth of 37% from 1991 (0.15 million) to 2011 (0.21 million), with a severely marginalized population due to the prevalence of extreme poverty, isolation from the mainland, and lack of organized support. The government compensation for post-flood housing restoration is insufficient to put forward the idea of 'building back better' (BBB) that is undoubtedly a priority for proactive adaptation to climate change.

## Method

**Figure 3:**
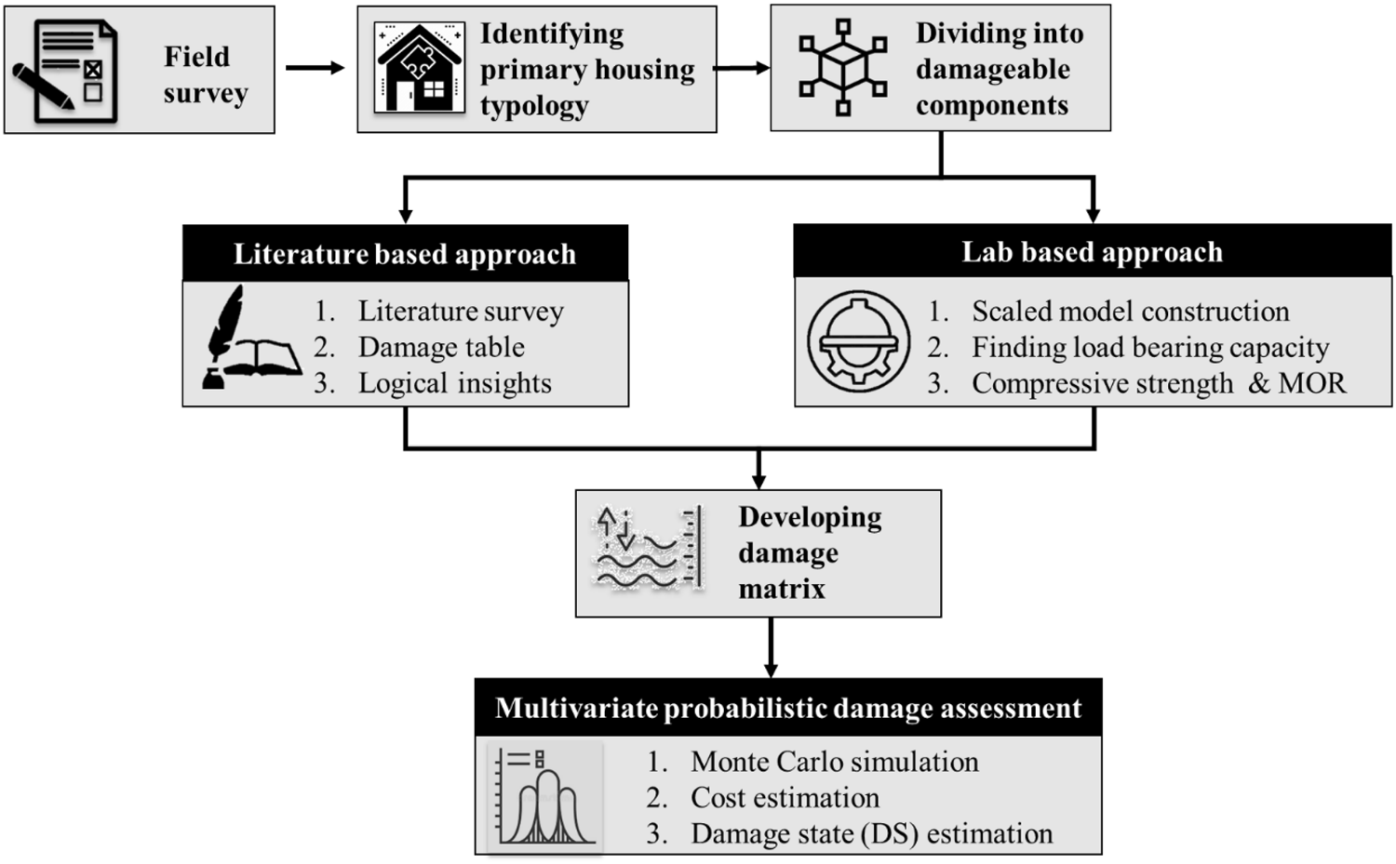
Methodology flowchart.

The paper estimates the damage behaviour of mud buildings based on a multivariate probabilistic technique using two pragmatic approaches as described in Fig. 3, (a) literature-based and (b) laboratory-based approach^50,51^. In the literature-based approach, the damage behaviour of mud building is marked using logical assumptions from the available scientific evidence. The laboratory-based method, however, evaluates the flood impact through real-time exposure created within an experimental setup. The damage behaviour proposed as a ‘multivariate damage matrix’ is an array of losses for the combined effect of flood depth and duration. The matrix developed using either of the proposed techniques was integrated into a probabilistic model to estimate the probable cost of coastal flood damage on Sagar Island.

### Building damage assessment based on damage matrix and cost simulation

The first phase of the work began with a field survey of the region and identification of primary housing typology along with its layout and essential details regarding its construction style, material (wall and roof), and overall building envelope. A standard building archetype was thus adopted for damage calculation. Following the archetype selection, the entire building was divided into components such as a plinth, floor, column, wall plaster, doors, and windows. The roof was excluded as it is usually not affected by flooding. The damage behaviour of the building components was supposed to be normally distributed between its upper and lower limit of resistance, where the mean was calculated using Eq. (1) and the standard deviation using Eq. (2)^52^.

The next step was to develop the multivariate damage matrix described elaborately in Sections "Literature-based approach for damage matrix" and "Laboratory-based approach for damage matrix". As with other damage curves, the matrix ranges from 0 to 1 (0 = no damage and 1 = complete damage). In mud buildings, the damage matrix of the dominant material (the mud wall section in this case) was assumed to reflect the failure pattern of all listed components. This is due to their significant contribution to altering stability and building repair costs compared to other materials. Using Monte Carlo simulations (MCs) with up to 1000 iterations, the depth and length of the flood were extrapolated. The values were spread randomly between a truncated normal distribution that ranges from the minimum and upto the maximum values of X_(min–max)_ and Y_(min–max)_ observed for the wall section. This distribution of depth and duration combinations was used to derive the flood loss potential curves (fragility curve) using both literature and lab-based approaches using Eq. (3). The loss potential is the probability of flood intensity exceeding the predefined resistance value or a damage stage (DS). The loss potential calculation process using the lab-based approach has been detailed out in Annexure 1.

The damage range is the ratio of the initial cost required for component repair/replacement. The entire building cost was estimated using the West Bengal schedule of rates. The total building damage cost for a particular flood event is the summation of the damage cost of all individual components (derived using Eq. 4). The building damage cost collected over extrapolated dataset is sliced into four stages within a ‘damage assessment table’ and represented as a three-dimensional axis using Eq. (5).

1 $$\mu =\frac{Xmin+Xmax}{2}$$

2 $$\sigma = \frac{Xmax-Xmin}{4}$$

3 $$Fr\left( r \right) = P\left[ {F\left( {x,y} \right) > R_{i} } \right]$$

4 $$DC=\sum_{i=1}^{n}a\cdot C{C}_{i}$$

5 $$Fr\left(DS\right)=P\left[DC>{C}_{t}\right]= nf/N$$

where $\mu$ = Mean resistance, $\sigma$ = Standard deviation, X = depth or duration value. (a) = Fragility function, P (F(x,y)) = Probability that flood character has a value x (depth), y (duration), Ri = Component resistance (value of i varies from 1 to 7). DC = damage cost of the entire building, CC = cost of an individual component, n = number of components in the building, a = loss ratio of the component, Fr (DS) = Fragility function for Damage state, ${C}_{t}$= damage cost stage (value of t varies from 0 to 5), DC = damage cost of the entire building.

### Literature-based approach for damage matrix

In this case, the material resistance of a building component acts as the key element for determining the degree of flood susceptibility. The material resistance is the range within which the disintegration and absolute failure of the component should take place due to its direct contact with floodwater. The start and end of this range were selected as the threshold values for the damage matrix. Mean and standard deviation values illustrate the intermediate level of damage. The threshold values for the duration were obtained per an exhaustive survey of scientific literature and reports that consisted of insight into post-flood field surveys, existing damage curves on mud buildings, and experiment data capturing the strength of mud blocks^28,41,53,54^. The same for flood depth varied between the level of direct contact and the component's complete submergence in floodwater. The following assumptions were considered for consistent execution of the damage matrix, (i) No significant damage when either depth or duration or both is zero,and (ii) failure of each component is independent of the other.

### Laboratory-based approach for damage matrix

This approach was employed to test the validity of the damage pattern adopted from literature-based reasoning. The experiment setup analyses the load-carrying capacity of the wall based on its structural strength when exposed to flood water. The carrying capacity is calculated using two sets of analyses (i) evaluation of compressive strength at given durations and (ii) evaluation of moment of resistance (MOR) for given depth and duration.

**Figure 4:**
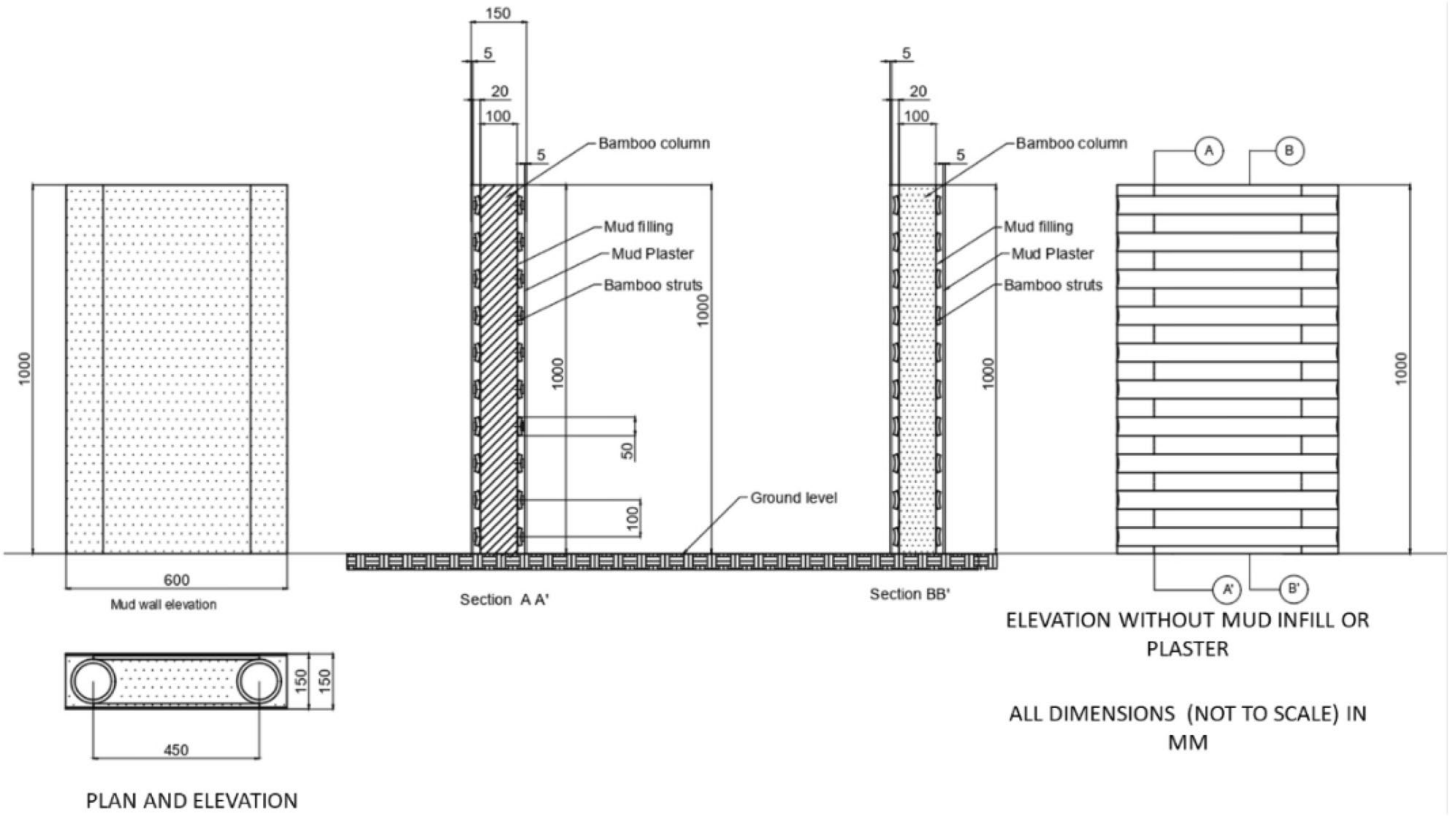
Wattle and daub wall cross-section for the experiment.

**Figure 5:**
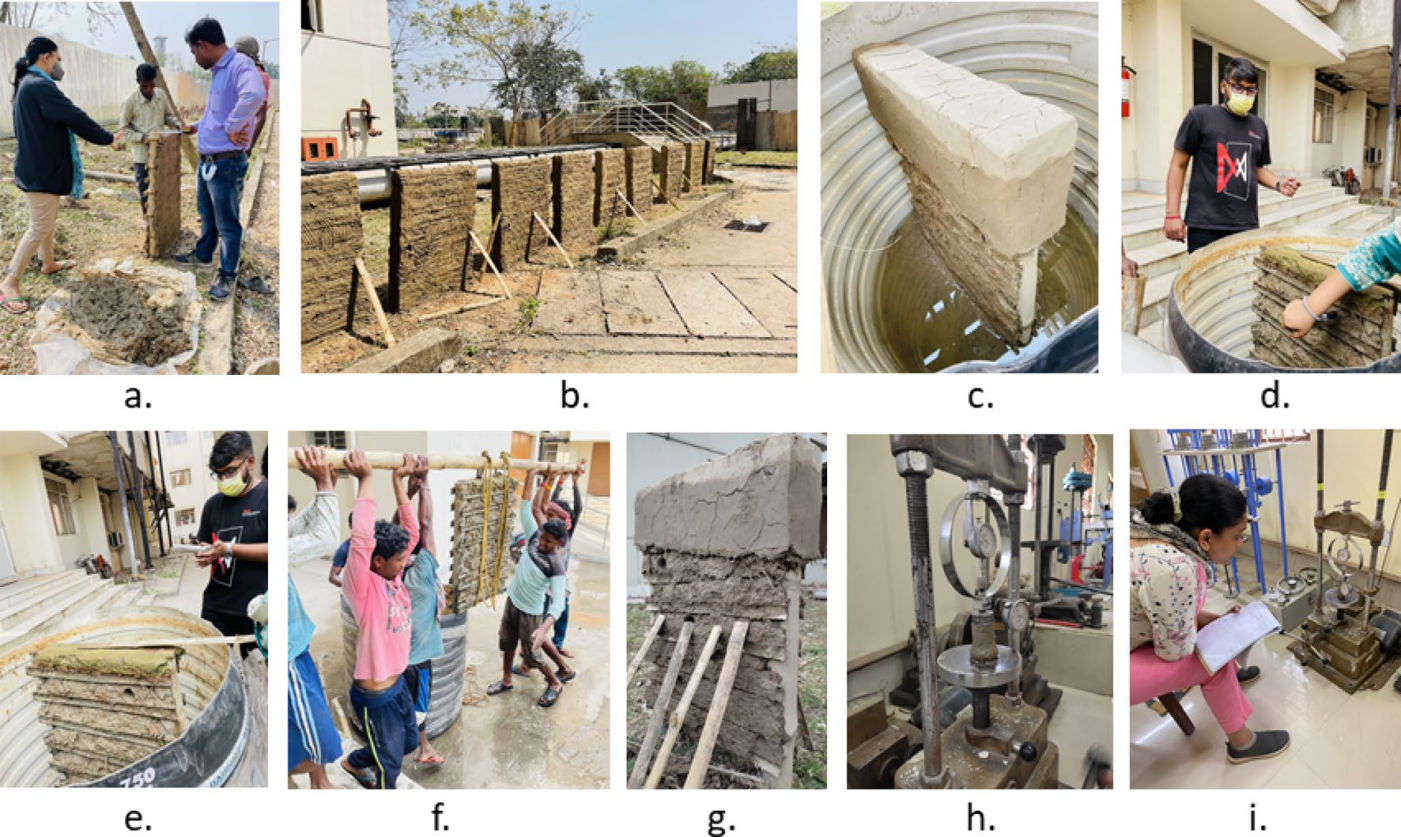
The experiment procedure.

The preparation of composite material for wall samples was based on native knowledge of the place. The walls consisted of a bamboo core filled with cob and later plastered with mud. They were sundried after preparation to ensure total dryness. Fifteen 1 m × 1 m wall specimens were constructed with 150 mm thickness to preserve the actual behaviour of wall panels post-inundation. Figure 4 represents the plan, elevation, and section for the wall specimen, and Fig. 5 depicts the course of the experiment. The specimens were immersed for 36 h, 72 h, 102 h, and 144 h at varying depths of 25%, 50%, 75%, and 100%. After the requisite immersion time had passed, the wet soil samples were extracted from the wall body for a compressive strength test. A cylindrical extractor of 76 mm in length and 38 mm in diameter was used, generating 3-sample from each wall specimen. The extracted specimens are subjected to structural procedures described in sections (a & b).

#### Evaluation of carrying capacity by compressive strength assessment at a given duration

The extracted sample from the wall post-inundation undergoes a destructive test called the ‘Unconfined Compressive Strength (UCS)’ test. The UCS test examines the “maximum axial compressive stress that a cohesive soil specimen can bear under zero confining stress”. The axial compressive strength of the soil is equal to the greatest load at which the failure occurs (along a diagonal plane or lateral building) divided by the cross-sectional area of the sample. The unconfined compressive test reading (using Eqs. 6 and 7) across the three samples was averaged to obtain a unique value for each duration.

If P_f_ is the failure load and A_f_ is the final cross-sectional area of the sample at failure,

6 $$q_{u} = \frac{{P_{f} }}{{A_{f} }}$$

7 $${\text{And A}}_{{\text{f}}} = \frac{{A_{0} }}{{\left( {1 - \varepsilon } \right)}}$$

where ${A}_{0}$ = the initial cross-sectional area, q_u_ = the axial compressive strength of a cohesive soil, ε = total deformation of the soil at failure.

Once the compressive strength was obtained, the load-carrying capacity of the wall was verified against the roof load using Eq. (10). The roof load calculation (from Eqs. 8 and 9) was based on the standard floor area dimension of 35 sq.m^55^ and tile roofing (tile roof wt. = 28–75 kg per sq. m) (IS 654 1992). The failure load for the wall section is determined for the given duration in Table 2.

Dead load of roof = total effective area of roof (Reff) X unit weight of the roof material (kg)

8 $${\text{R}}_{{{\text{eff}}}} = 2\left( {\frac{1}{{2{ }}}bh + \frac{1}{{2{ }}}\left( {l1 + l2} \right)h} \right){ } \cdot { }unit\; { }load{ }\; of\; { }roof$$

Cross-section area of wall/ effective area under loading (sq.m),

9 $${\text{W}}_{{{\text{eff}}}} \; = \;\left( {A1XA2 - B1XB2} \right)$$

10 $${\text{Carrying capacity of the wall }}\left( {{\text{kg}}} \right) = \left( {q_{u} { } \times { }W_{eff} } \right)$$

where ($${q}_{u})$$ = Compressive strength.

#### Evaluation of carrying capacity by the MOR assessment at a given depth and duration

The effect of water depth on the wall cross-section was estimated based on the moment of resistance. The compressive strength for each duration from Eq. (6) (36 h, 72 h, 102 h, and 144 h) was used to compute the MOR for each effective length (1575 mm, 1050 mm, and 525 mm) using Eq. (11), adapted from the RCC section without reinforcement^56^. The effective length is the unsubmerged portion of the wall. The total wall length is the standard length observed in the mud housings, i.e., 2100 mm. The MOR developed for each combination of depth and duration was compared with the moment generated due to roof load (using Eq. 12, C = roof load) to identify the point of failure.

11 $${\text{Moment of resistance }}\left( {{\text{M}}_{{{\text{or}}}} } \right) \, \left( {{\text{kN}} - {\text{m}}} \right) = \left( {C \cdot Z} \right)$$

where M_or_ = Moment of resistance, C or ($$q_{u} )$$ = Compressive strength, Z = Lever arm;

Now, Z = d (d−n/3) where d = Effective length of the (unsubmerged) wall section, n = distance of the neutral axis from the extreme fiber = 0.5d.

Hence, it will not be wrong to conclude that the wall section suffers subsequent loss until the final failure. The ‘failure matrix’ captures this subsequent loss at each point of depth-duration by taking the weighted average of the normalized value of compressive strength and moment of resistance (from Eq. 12) obtained from the experimental observations.

12 $$Damage\;\left( D \right) = 0.5\;\left( {M + C} \right)$$

where M = Moment of resistance, C = Compressive strength.

## Results and discussion

### Literature-based approach

The census data certifies mud housing as the dominant housing typology comprising 75% of the total built-up in Sagar. Figure 1 provides a glimpse of “wattle and daub” construction prevalent in the case area. Hence, the prototype adopted for the study replicates the commonly occurring single-story dwelling unit of 35 sq.m. The prototype was then divided into six components, namely plinth, floor, wall plaster, wall, door, and window, for component-based damage analysis. Each component was allocated a damage threshold (lower and upper limit) based on its minimum and maximum resistance to flooding (depth and duration), as shown in Table 1. Mud wall was chosen to develop the flood damage matrix, given its maximum contribution to overall building cost. The presence of a bamboo core inside the wall was neglected due to the absence of supporting literature.

**Table 1:** Damage table of mud housing in Sagar.

Para-meters	Threshold	Building components susceptible to flooding						
		(1)	(2)	(3)	(4)	(5)	(6)	(7)
Depth in mm	Min	0.01	0.3	0.45	0.01	0.45	0.45	1.05
	Max	0.3	0.45	2.5	2.5	2.5	1.8	1.65
	Avg ($\mu )$	0.155	0.375	0.225	1.055	1.275	1.125	1.35
	Std. dev. ($\sigma )$	0.0725	0.0375	0.1125	0.5225	0.4125	0.3375	0.15
Duration in hrs	Min	2	2	24	2	2	24	24
	Max	38	38	48	38	38	48	48
	Avg ($\mu )$	20	20	36	20	20	36	36
	Std. Dev. ($\sigma )$	9	9	6	9	9	6	6
	Cost (Rs)	4306	822.5	2000	11,341	15,968	2000	3500

1- Brick and mud plinth, 2- mud floor, 3- bamboo column, 4 – mud plaster, 5- mud wall, 6 -wooden door, 7- wooden window.

**Figure 6:**
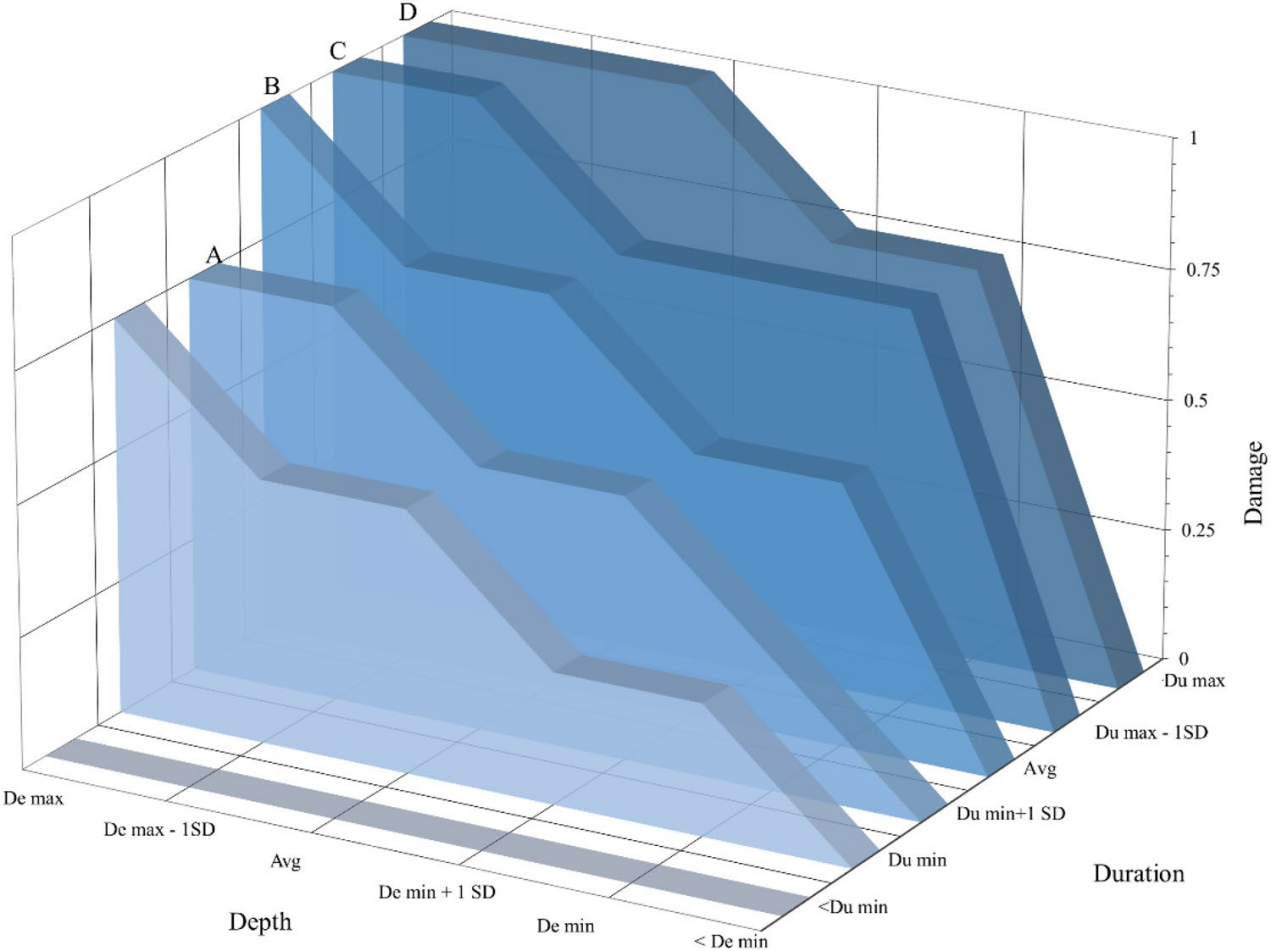
Building damage matrix from the literature-based approach.

Figure 6 showcases the building damage behaviour for mud walls in terms of loss ratio. It presents the damage value based on the combination of depth and duration. The X-axis in the proposed matrix is for depth values, and the Y-axis is for duration. The probable damage on the Z-axis is as per the logical reasoning acquired from the literature. The literature review suggests a subsequent loss in material strength with increasing flood intensity^54,57^, meaning that the prolonged inundation enhances the soil particle saturation, undermining their cohesive properties. Since the material cohesion is inversely related to the cross-sectional area, stiffness, and overall load-bearing capacity of the structure, the inundated area, cannot provide adequate support against the roof load or the moment generated. Therefore, the wall may crumble if the effective length (non-inundated length) is not sufficient to resist the roof load.

The depth and duration are divided into six segments (below the minimum to maximum). The values have been derived from Table 1. Below the minimum segment is when the flood depth or duration is within the structural resistance limit of the building component and hence does not cause damage. Beyond the minimum segment, the actual deterioration starts until it fails at the maximum point. The first row (< De min to < De max) showed no damage since the zero-depth meant no direct contact of the building with the flood water. Similarly, the duration at these points is insignificant enough to affect the building strength.

At point A, the depth value is noted as 1SD beyond the minimum flood level and duration is still 1SD below the maximum; the wall suffers 75% damage due to the effect of depth. The water depth was assumed to have a comparatively larger effect than duration due to the correlation between the MOR and effective wall length, where MOR is the square of the wall length^56^. Hence, the wall is likely to fail in complete submergence at points B, C, and D, as shown in Fig. 6.

### Laboratory-based approach

The lab-based approach analyzes compressive strength for all chosen values of durations, as presented in Table 2. The R-square (0.97) depicts significant reliability of the relationship; hence, Eq. (13) is extrapolated to obtain the intermediate value of the compressive stress. The compressive stress at 0 duration (negligible contact with water) is 12.48 kPa. It is also noted that the experimentally calculated value for a dry sample was 1127.740 kPa.

13 $$q_{u} = - 0.0{\text{322D}} + {12}.{488}$$

where ${q}_{u}$ = compressive strength (kPa), D = duration (hrs.)

**Table 2:** Compressive strength and load-carrying capacity of wall sections for each duration.

Duration (Hours)	Compressive strength (kPa)	Load carrying capacity of wall (Kg)
0	12.488	4533.30
36	11.5714	4200.65
72	9.8422	3572.93
102	9.1253	3312.68
144	7.9969	2903.04

**Figure 7:**
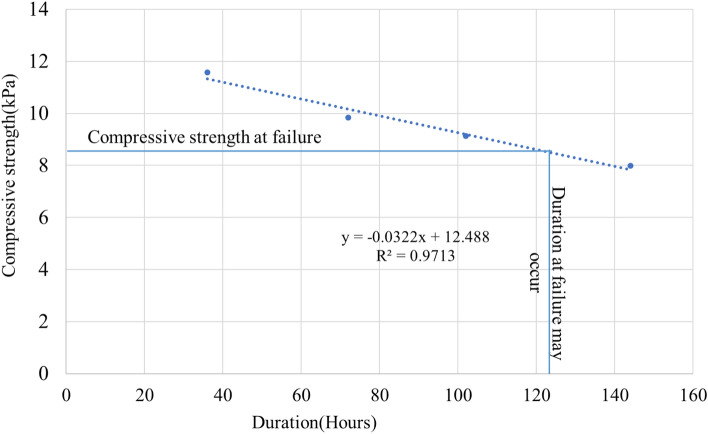
Compressive strength at different flood durations.

The load-carrying capacity of the wall was tested against the estimated roof load of 3107 kg, that exerts stress of 8.57 kPa. The load-carrying capacity of the wall at zero duration is 4533 kg, sufficiently larger than the roof load. The wall will likely fail at any duration beyond 121.67 h as the carrying capacity at this point is equal to the roof load (as shown in Fig. 7).

The compressive strength was used to evaluate the MOR, which is the most crucial parameter for estimating the failure depth across the wall cross-section. The MOR for an unsubmerged, effective length of 2100 mm due to roof load is 3.44 kN-m. Table 3 consists of the MOR threshold for the changing values of effective length against roof load. The threshold values identify the maximum duration after which the wall may succumb due to bending moment exceedance.

**Table 3:** MOR due to roof load for various wall sections.

Effective wall length (mm)	Flood depth w.r.t effective length (%)	Width (mm)	Comp (N/sq. m)	MOR (kN-m) threshold
2100	0	150	8.57	3.44
1575	25	150	8.57	1.3286
1050	50	150	8.57	0.5905
525	75	150	8.57	0.1476

**Figure 8:**
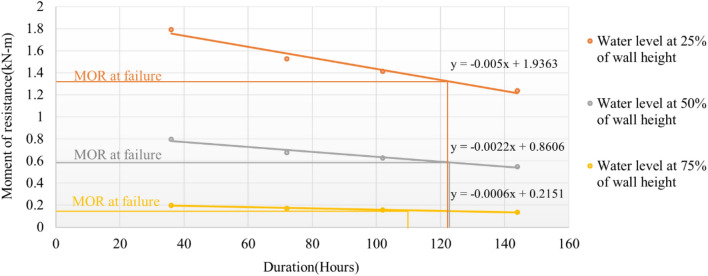
Failure duration corresponding to MOR.

Table 4 presents the MOR estimated corresponding to the compressive strength from Table 2. The relationship has been extrapolated with respect to the effective length to identify the failure duration using MOR threshold represented in Fig. 7. Hence, the failure duration noted for the effective length of 1575 mm, 1050 mm, and 525 mm are 122 h, 118 h, and 111.83 h, respectively. This denotes that the mud building may observe sudden collapse when the flood duration exceeds the threshold value. The damage matrix proposed in Fig. 8 highlights the inferences of damage behaviour derived during the experiment.

**Table 4:** Threshold value of MOR estimated using the compressive strength.

Duration (hrs.)	Comp (N/sq. mm)	Width (mm)	Effective wall length (mm)	MOR (kN-m)
36	0.0115	150	1575	1.7938
			1050	0.7972
			525	0.1993
72	0.0098	150	1575	1.5255
			1050	0.6780
			525	0.1695
102	0.0091	150	1575	0.7072
			1050	0.3143
			525	0.0785
144	0.0079	150	1575	0.6193
			1050	0.2752
			525	0.0688

**Figure 9:**
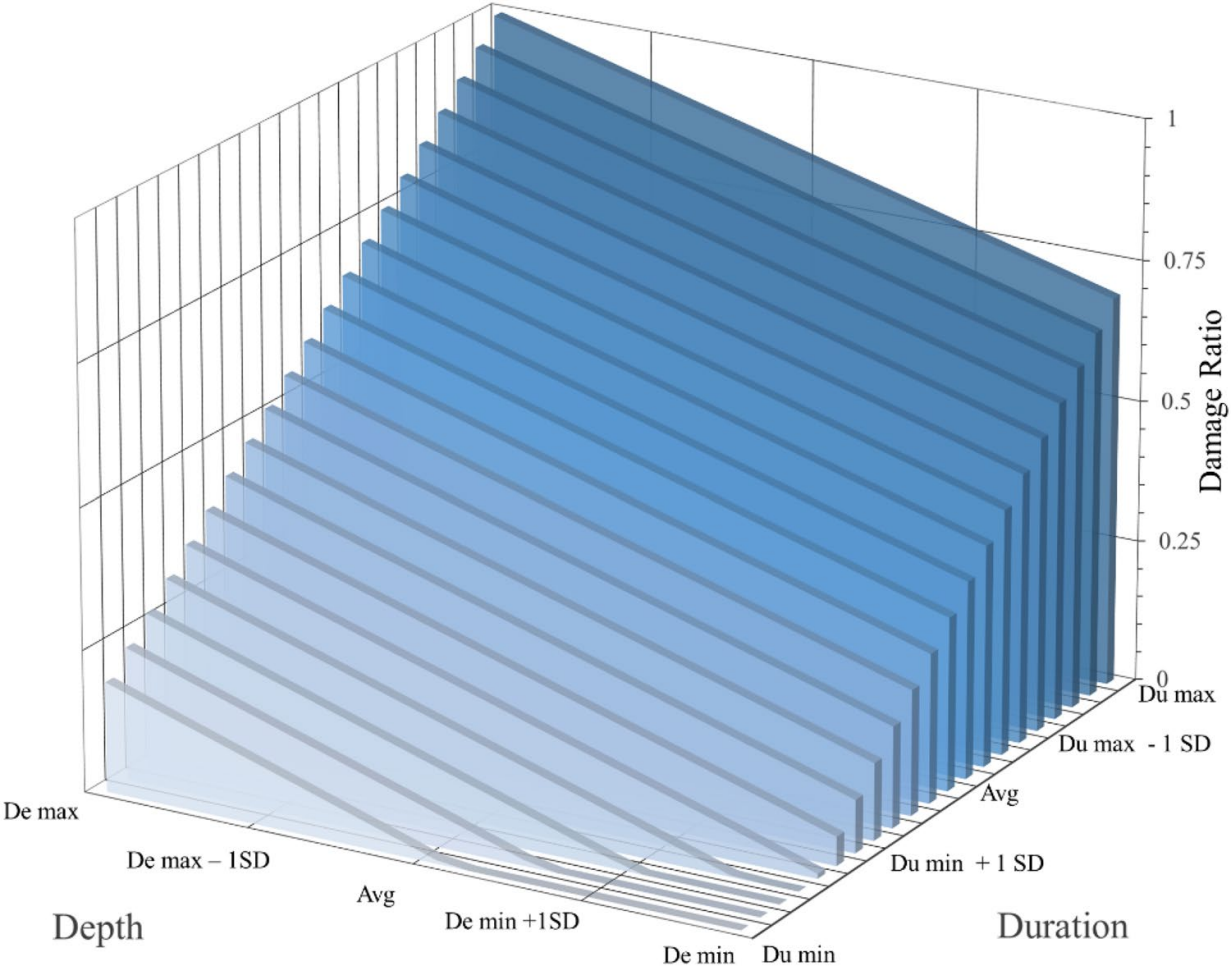
Building damage matrix from the experiment-based approach.

The experimentally derived damage matrix (Fig. 9) validates the linear damage trend associated with the depth and duration variables. It also clearly reveals the prominence of flood duration, which has been relatively overlooked in the prior analysis of flood impact on mud buildings. The matrix shown in Fig. 9 has been developed by averaging out the depth and duration effects (from Eq. 12). It illustrates the intermediate layers of damage between Duration (Du) min and Du max at an interval of every 6 h. Du minimum can be any significant value of flood duration, say 1 h; therefore, the damage at this stage rises concerning flood depth alone. Similarly, even with a minimum depth, the wall will suffer damage up to 75% due to prolonged hours of duration. The matrix also validates the dominant effect of flood depth which is almost twice the duration in undermining the stability of mud buildings. The workability relative to the literature-based approach is analyzed in the following section.

### Development of final damage stage

**Figure 10:**
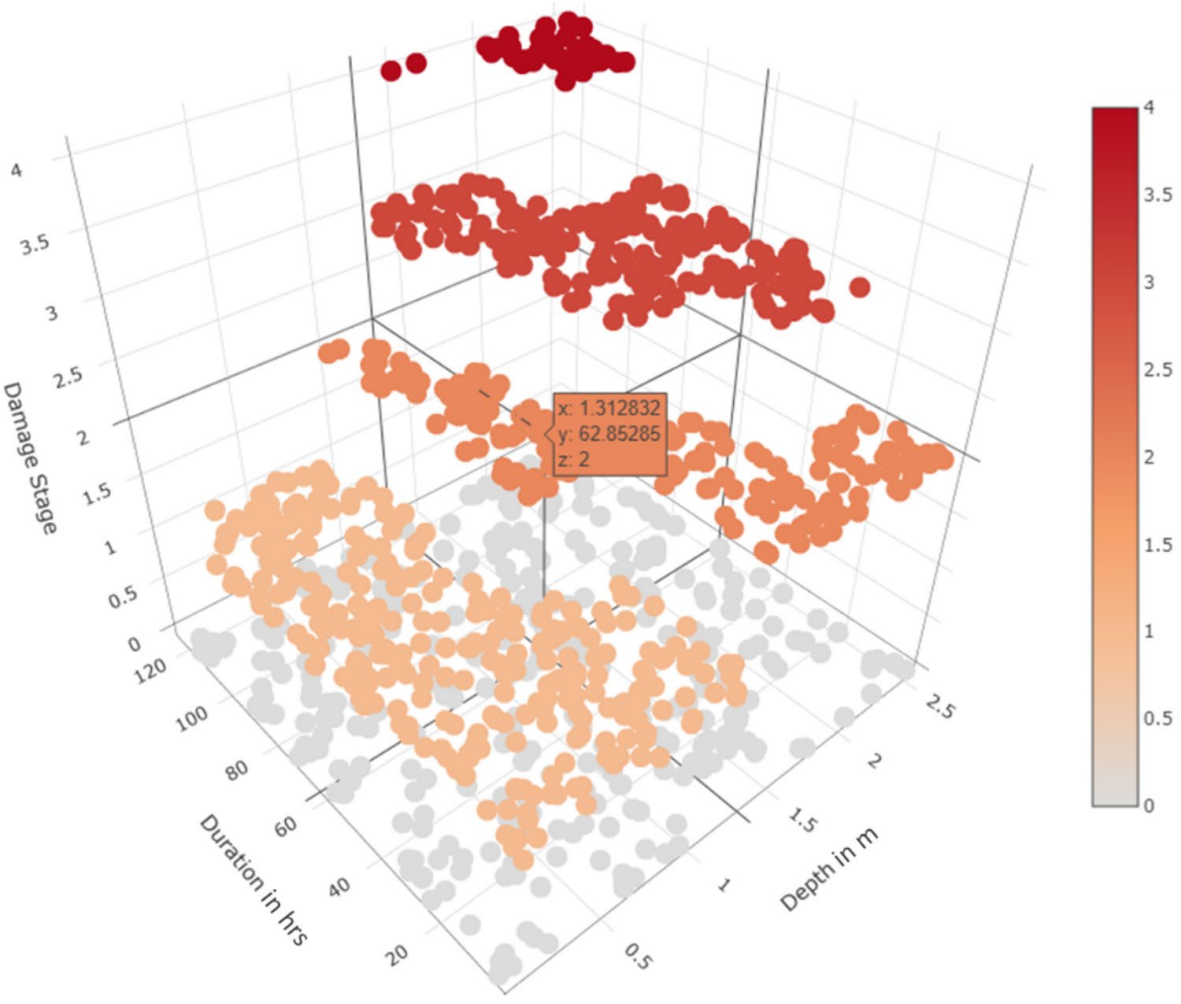
Damage stage for mud building based on the experiment-based approach.

**Figure 11:**
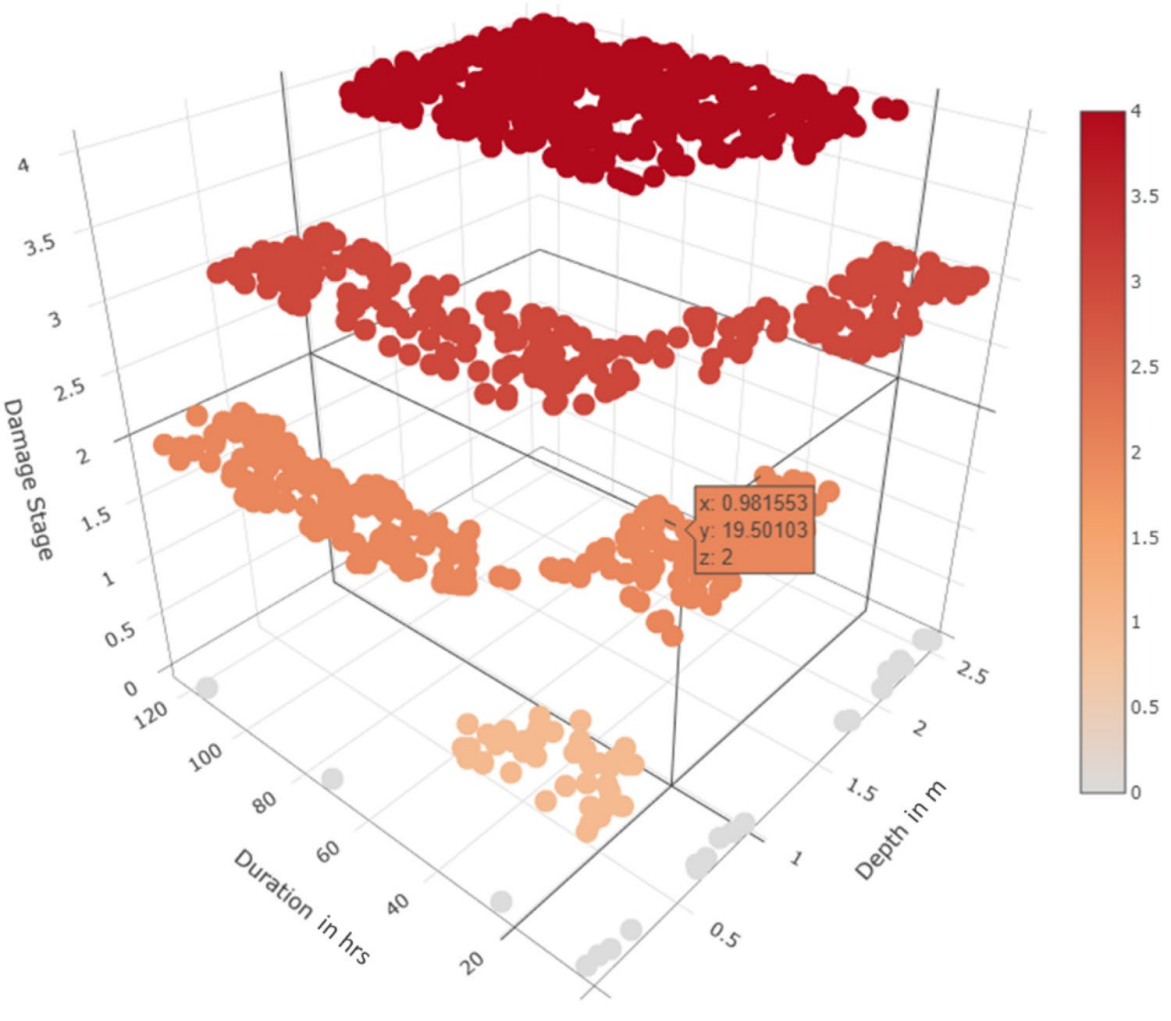
Damage stage for mud building based on the literature-based approach.

After developing the multivariate damage matrices from the proposed lab-based and literature-based techniques, their applicability needs to be tested using synthetically generated flood data values. Montecarlo simulations were employed to generate a 1000 combination of flood depth and duration. The values generated were analysed through a component-wise damage assessment process using the proposed matrices. The probable building damage in terms of cost was estimated for each combination of depth and duration. The buildings were then placed into four defined Damage Stage (DS) categories where DS1 refers to 0 to 25%, DS2 is 25% to 50%, DS3 is 50 to 75% and DS4 is 75–100% loss of the total building cost. A step-by-step process for the calculation of probable loss and related ‘cost assessment’ is shared in Annexure 1 to provide further insight. The Damage Stage graph of probabilistically simulated flood data employing both lab-based and literature-based approaches is presented as Figs. 10 and 11. The X and Y-axis in the graph represent flood depth and duration, while the Z-axis represents the Damage Stages that can be perceived as the proportion of the total cost of the building required for repair in a post-flood scenario. Although developed for a common objective of estimating multivariate damage associated with mud buildings, the literature-based approach overestimates the damage calculation by 9.5 times in the case of DS4 and underestimates the same by 5.5 times for DS1. This is due to the absence of a precise value of failure duration which is impossible to be obtained from empirical data that continues to be the basis of existing literature. Hence, the structural analysis allows the continuous nature of the lab-based approach feasible for extracting the absolute cost of damage, while the literature-based approach remains tentative. Needless to say, the lab-based approach maintains high precision and accuracy; however, in a resource-constrained environment, the literature-based method shall meet the cause if equipped with adequate information regarding failure thresholds.

The matrices developed can be used to estimate near real-time scenarios by taking the example of the flooding during cyclone Yaas in 2021. The water level in Sagar Island during cyclone Yaas reached around 5.85 m and 7.5 m on May 26th and 27th, respectively. Hence, the flooding level beyond the high-water line (at 5.02 m for Sagar) was estimated as 1.21 m and 2.86 m. Hence, the local elevation values (using the Digital Elevation Model (DEM) of Sagar shall be subtracted from the flood level to attain depth values corresponding to each building in Sagar. The duration values as 24-h and 48-h shall be chosen to estimate the probable loss for Sagar Island using the proposed damage matrix. Consequently, the developed approach turns out to be a state-of-art solution for flood loss estimation.

## Conclusion

The absence of predefined damage behaviour or curves is the major bottleneck in estimating flood impact on the mud building typology in developing countries. Moreover, the climate change induced rise in the MSL makes damage assessment a non-negotiable process for low-lying coastal regions subjected to prolonged flooding amidst a resource-constrained environment. The current research thus proposes two approaches (a) literature based and (b) lab-based approach using a multivariate-probabilistic technique to develop a mud building damage matrix. The damage matrices developed are used to calculate the probable cost of flooding and associated Damage Stage for probabilistically obtained depth duration value from the Montecarlo simulations. The lab-based being one of its kind, is duly accurate given the continuous nature of observation and precise estimation of flood duration, which is a crucial data limitation in the literature-based damage assessment process. The experimental analysis reveals over 90 percent reduction in the strength of the mud wall with mere contact with the flood water. Hence, future housing policies may consider integrating waterproofing as a suitable strategy for climate resilience of the native housing typology that comprises 34% of the housing stock in India.

The proposed multivariate damage matrix can be repeated over diverse housing typologies. Although, complex housing with multiple rooms and larger variations in building material would mean complicated integration of their behaviour derived using either of the two approaches. Since, the overall focus of the current study remains on the mud-building damage behaviour that can vary based on soil preparation and construction technique; the proposed approach can be modified accordingly to act as a testimony of building resilience. The flexibility of multivariate matrix over spatial and temporal dimensions is well proven; however, it demonstrates epistemic uncertainties by choosing an archetype to represent the entire building fraternity in Sagar, which may also be the scope of future research. Finally, the tool developed shall prove pivotal in assessing the cost–benefit for building and spatial-level solutions for resilient development. The prospective research shall entail the application of the proposed matrix in evaluating the efficacy of nature-based solutions in reducing flood impact on the built environment.

## Supplementary Information

Supplementary Information.

## Data Availability

The datasets used and/or analysed during the current study available from the corresponding author on reasonable request.
