# Supplementary material for: Developing assessment flow for damage estimation of mud housing typology through a case study against coastal floods
Source: Sci Rep. 2023 Apr 25;13:6712. doi: 10.1038/s41598-023-33468-6 (PMC10130091; doi:10.1038/s41598-023-33468-6)
Supplement: Supplementary file 1 — Supplementary Information. [file 41598_2023_33468_MOESM1_ESM.docx]

**Annexure 1**

Methodology for developing the experimentally derived building damage stage graph as shown in Figure 10

The experimentally derived damage stage graph is an application of a damage matrix used to estimate the damage cost of a building at a given depth and duration. Therefore, the damage matrix shown in Figure 8 is assembled into a tabular format as shown below (Table 5). The flood duration column in the table consists of the normally distributed duration values for failure of the mud wall. Similarly, the flood depth is represented across the first row. The resulting damage from the depth and duration combination is filled based on the experimental observations. For instance, the loss recorded at the mean depth and duration is around 0.42. In this paper, the mud wall damage matrix has been universally adopted to denote the building behaviour of the other components present in the building. However, the failure threshold for their depth and duration has been taken from the prominent literature available on building materials as shown in table 1. It is relevant to mention that the duration threshold (in Table 5) considered for the mud wall is different than in Table 1 as it was replaced post-experiments.

To test the efficacy of the damage matrix, Monte Carlo simulations are used to generate the random values of flood depth and duration, as shown in table 6 (columns a and b). The random depth and duration values are assigned the damage ratio based on table 5 using an index & match’ formula available in MS Excel. The damage ratio assigned to each building component is multiplied by its corresponding cost. Finally, the sum of the total cost across the flood iteration is checked to determine the range of failure. The range is divided into five cost intervals from DS0 to DS4, as shown in Figure 9.

Table 5: Component damage table for mud wall

|  | Flood Depth | Min | Std. dev.,(σ) | Avg (μ) | Std. dev.,(σ) | Max |
| --- | --- | --- | --- | --- | --- | --- |
| Flood duration | Threshold | 0.45 | 0.9625 | 1.425 | 1.9375 | 2.5 |
| Min | 1 | 0 | 0.03 | 0.25 | 0.46 | 0.67 |
| Std. dev.,($\sigma)$ | 30.5 | 0 | 0.12 | 0.33 | 0.54 | 0.75 |
| Avg ($\mu)$ | 61 | 0.08 | 0.21 | 0.42 | 0.62 | 0.83 |
| Std. dev.,($\sigma)$ | 92.5 | 0.18 | 0.3 | 0.5 | 0.71 | 0.91 |
| Max | 122 | 0.28 | .40 | .60 | .80 | 1 |

Table 6: Primary cost assessment table

|  |  |  | Plinth | Flooring | Column | Wall plaster | Wall | Door | Window | Total cost | Damage Stage (DS) |
| --- | --- | --- | --- | --- | --- | --- | --- | --- | --- | --- | --- |
|  |  | (1) | 4306 | 822.5 | 2000 | 11341 | 15968 | 2000 | 3500 | 39937 |  |
|  | (a) | (b) | DR1 | DR2 | DR3 | DR4 | DR5 | DR6 | DR7 | Σ(1 x 2) |  |
| Sim | Dep | Dur |  |  |  |  |  |  |  |  |  |
| 1 | 1.43 | 77.23 | 0.83 | 0.83 | 1 | 0.42 | 0.42 | 1 | 1 | 23226.45 | 3 |
| 2 | 0.31 | 79.20 | 0.83 | 0 | 0 | 0 | 0 | 0 | 0 | 3573.98 | 1 |

(1) = cost of each of the component

DR = damage ratio added from table 5 in reference to threshold values from table 7

Table 7: Flood damage threshold of building components

|  | Plinth | Flooring | Column | Wall plaster | Wall | Door | Window |
| --- | --- | --- | --- | --- | --- | --- | --- |
| Material | Mud | Mud | Bamboo/timber | Mud | Mud | timber | timber |
| Cost (in Rs) | 4306 | 822.5 | 2000 | 11341 | 15968 | 2000 | 3500 |
| Depth threshold (in mm) | | | | | | | |
| Min | 0.01 | 0.3 | 0.45 | 0.45 | 0.45 | 0.45 | 1.05 |
| Std. dev.,($\sigma)$ | 0.082 | 0.33 | 0.96 | 0.96 | 0.96 | 0.78 | 1.2 |
| Avg ($\mu)$ | 0.155 | 0.370 | 1.42 | 1.42 | 1.42 | 1.12 | 1.35 |
| Std. dev.,($\sigma)$ | 0.192 | 0.41 | 1.93 | 1.93 | 1.93 | 1.46 | 1.5 |
| Max | 0.30 | 0.45 | 2.5 | 2.5 | 2.5 | 1.8 | 1.65 |
| Duration threshold (in hrs) | | | | | | | |
| Min | 1 | 1 | 24 | 1 | 1 | 24 | 24 |
| Std. dev.,($\sigma)$ | 30.5 | 30.5 | 30 | 30.5 | 30.5 | 30 | 30 |
| Avg ($\mu)$ | 61 | 61 | 36 | 61 | 61 | 36 | 36 |
| Std. dev.,($\sigma)$ | 92.5 | 92.5 | 42 | 92.5 | 92.5 | 42 | 42 |
| Max | 122 | 122 | 48 | 122 | 122 | 48 | 48 |
